# A Spatial Consistency-Guided Sampling Algorithm for UAV Remote Sensing Heterogeneous Image Matching

**DOI:** 10.3390/s26010102

**Published:** 2025-12-23

**Authors:** Runjing Chen, Haozhe Lv, Jiaxing Zhou, Zhigao Chen, Taohong Li, Xinping Zhang, Yunpeng Li, Zhibin Zhan

**Affiliations:** 1 School of Computer and Information Engineering, Xiamen University of Technology, Xiamen 361024, China; chenrj@xmut.edu.cn; 2 School of Electrical Engineering and Automation, Xiamen University of Technology, Xiamen 361024, China; 2510606130@stu.xmut.edu.cn (H.L.); 2422141004@stu.xmut.edu.cn (Z.C.); 2510606129@stu.xmut.edu.cn (T.L.); 2510606124@stu.xmut.edu.cn (X.Z.); 2510606104@stu.xmut.edu.cn (Y.L.); 2510606102@stu.xmut.edu.cn (Z.Z.); 3 Xiamen Key Laboratory of Frontier Electric Power Equipment and Intelligent Control, Xiamen 361024, China

**Keywords:** heterogeneous images, image matching, spatial consistency, triplet relationships

## Abstract

In UAV visual localization applications, the quality of image matching directly affects both the precision and reliability of the visual localization task. In UAV visual localization tasks, high-resolution remote sensing images are typically used as reference maps, whereas UAV-acquired aerial images serve as real-time inputs, enabling the estimation of the UAV’s spatial position through image matching. However, due to the substantial difference in imaging mechanisms and acquisition conditions between reference and real-time images, heterogeneous image pairs often contain numerous outliers, which significantly hinder the direct application of traditional matching algorithms such as RANSAC. To address these challenges, a spatial consistency-guided sampling algorithm is proposed. First, the initial correspondences are constructed based on triplet relationships, and their structural features are subsequently extracted. Then, a minimal subset sampling strategy is developed to improve sampling efficiency. Next, a data subset refinement strategy is introduced to further improve the robustness of sampling. Finally, extensive comparative experiments are conducted on the University-1652 and DenseUAV public datasets against several state-of-the-art feature matching algorithms. The experimental results demonstrate that the proposed algorithm achieves superior performance in correct matching rate, substantially enhancing the matching performance in heterogeneous image matching. Moreover, the proposed algorithm requires approximately 0.15 s per matching on average, and while maintaining the highest matching accuracy, it exhibits significantly higher computational efficiency than advanced sampling algorithms such as TRESAC and RANSAC, demonstrating strong potential for real-time applications in UAV visual localization tasks.

## 1. Introduction

With the rapid advancement of spatial information technologies, unmanned aerial vehicles (UAVs) have emerged as indispensable tools in remote sensing. Owing to their exceptional maneuverability, flexibility, stealth, cost-effectiveness, and strong information acquisition capabilities, UAVs have attracted widespread attention and found broad applications across multiple domains. UAV image processing, analysis, and recognition technologies have been extensively employed in various fields, including search and rescue [1], terrain mapping [2,3] and target localization [4]. The rapid advancement of these technologies has significantly driven progress in image processing, computer vision, and pattern recognition, establishing them as pivotal frontiers in contemporary research. Nevertheless, realizing autonomous mission execution on UAV platforms remains fundamentally constrained by the challenges of high-precision localization and reliable navigation. At present, UAV localization and navigation primarily rely on multiple navigation systems. The Global Navigation Satellite System (GNSS) provides high localization accuracy [5]; however, it often fails in complex environments due to electromagnetic interference or signal occlusion. Inertial Navigation Systems (INSs) operate autonomously without reliance on external signals and achieve high short-term accuracy [6]; nevertheless, cumulative errors degrade performance over time. Radio navigation estimates the UAV’s relative position through the transmission and reception of electromagnetic signals, but it is similarly susceptible to external interference. Dead reckoning estimates the UAV’s real-time position using onboard sensor data and wind information; however, its accuracy is limited by measurement precision, leading to error accumulation over time. Enhancing the reliability of UAV navigation necessitates the integration of multi-source navigation techniques and the exploration of advanced fusion strategies to cope with the diverse challenges encountered in complex environments.

As image sensor and image processing technologies continue to advance, the integration of computer vision and artificial intelligence into UAV localization and navigation has become progressively more prevalent [7]. Modern UAVs are typically equipped with image sensor systems and AI algorithms, which ultimately leads to a comprehensive enhancement of UAVs’ localization and navigation performance. Image-sensor-based visual navigation can generally be categorized into map-based and mapless methods, depending on whether prior maps are utilized [8]. In map-based navigation, real-time images are matched with pre-stored reference maps to estimate the UAV’s position, e.g., scene matching. In contrast, mapless navigation relies on motion estimation derived from image sequences, estimating the UAV’s relative motion and pose by analyzing inter-frame feature variations. Techniques such as visual odometry (VO) [9] and simultaneous localization and mapping (SLAM) [10] play a pivotal role in enhancing the autonomous navigation capabilities of UAVs.

Image-sensor-based visual localization and navigation technologies demonstrate substantial application potential. These technologies can achieve localization accuracy comparable to GNSS and provide effective alternatives when GNSS signals are degraded or unavailable, thereby reducing UAV’s dependence on external resources and offering significant engineering and practical value [11]. However, without external constraints, visual odometry and SLAM methods accumulate errors over time, thereby limiting their ability to achieve reliable global navigation independently [12,13]. In contrast, scene-matching-based navigation techniques effectively mitigate error accumulation, offering a more stable and reliable navigation solution. Typically, scene-matching-based navigation techniques pre-store satellite remote sensing images as reference maps on the UAV and capture real-time images through onboard sensors during flight, which are subsequently matched with the reference maps. Since the geographic coordinates of the reference images are precisely known, successful matching enables the determination of the UAV’s absolute position, thereby serving as an auxiliary localization approach to complement GNSS and INS [14]. UAV platforms typically employ satellite remote sensing images as reference maps. These images provide extensive spatial coverage and are readily accessible; however, their lengthy production cycles result in considerable discrepancies from UAV real-time images in terms of acquisition time, season, illumination, sensor characteristics, and imaging altitude, thereby imposing stringent requirements on the robustness and adaptability of image matching algorithms. Consequently, extensive research efforts have been devoted worldwide to addressing the critical challenge of heterogeneous image matching [15,16,17].

Feature matching represents a fundamental stage in scene matching, aiming to establish spatial geometric correspondences between images based on extracted feature points and to achieve spatial alignment and pose estimation through geometric model fitting. Consequently, robust model fitting plays a pivotal role in reliable feature matching. However, satellite and UAV images exhibit significant heterogeneity arising from distinct imaging mechanisms, such as variations in resolution, viewing angle, and illumination. These discrepancies often introduce numerous outliers in the extracted features. When the model fitting algorithm lacks robustness, the estimated parameters can be severely biased, impeding effective outlier rejection, generating incorrect spatial correspondences, and ultimately degrading localization accuracy.

The effectiveness of robust model fitting methods largely depends on the design of their underlying sampling strategies. Random Sample Consensus (RANSAC) is a classical and representative paradigm that remains widely adopted in feature matching missions [18]. RANSAC iteratively samples minimal subsets from the input correspondences to generate multiple model hypotheses, selecting the one exhibiting the highest structural consistency as the final estimated model. The minimal subset represents the smallest collection of data points required to instantiate a model hypothesis. Under ideal conditions, RANSAC aims to sample subsets composed entirely of inliers, thereby yielding robust model parameters and minimizing the influence of outliers. However, the number of required RANSAC iterations increases exponentially with rising outlier ratios and larger minimal subset sizes. When the inlier ratio is low, obtaining an all-inlier subset typically requires a large number of iterations, thereby significantly reducing computational efficiency [19].

To enhance the performance of robust model fitting, numerous RANSAC variants have been proposed in the literature. PROSAC improves sampling efficiency via a progressive strategy guided by prior matching quality [20]; LO-RANSAC performs local optimization after model generation to enhance accuracy [21]; and MLESAC and MSAC introduce statistical criteria to refine model evaluation [22]. These methods achieve a reasonable trade-off between efficiency and robustness, leading to their widespread adoption in image feature matching tasks. However, their performance remains strongly influenced by sampling efficiency and the inlier ratio. When the outlier ratio is high or the geometric structure becomes complex, convergence deteriorates markedly, and model fitting may even fail.

To overcome the limitations of RANSAC, a variety of advanced guided sampling algorithms have been proposed [23,24,25]. In studies based on guided sampling strategies, exploiting local geometric relationships among feature points to enhance spatial consistency has become an important research direction. These algorithms leverage prior information, such as pairwise relationships and residual ordering. Pairwise relationships are widely used as a metric for inlier discrimination, based on the assumption that inliers tend to be spatially proximate while outliers are randomly distributed, thereby improving the robustness and efficiency of the sampling procedure. However, outliers may occasionally exhibit local geometric characteristics similar to those of inliers, which introduces a risk of misclassification when relying solely on pairwise relationship-based discrimination. Consequently, relying solely on pairwise relationships during sampling is often insufficient to effectively distinguish inliers from outliers, thereby reducing the robustness of model fitting.

Representative methods such as TRESAC [26] generate model hypotheses by randomly sampling triplets, with the core idea that triplets impose stronger geometric constraints and thus increase the probability of rapidly identifying all-inlier subsets. In such methods, triplets are typically treated as “sampling seeds,” and their effectiveness largely depends on the probability of randomly selecting a clean triplet. When confronted with the large number of non-uniformly distributed outliers commonly present in heterogeneous image matching scenarios, the sampling efficiency and robustness of these methods may still degrade. More importantly, TRESAC places limited emphasis on actively and structurally selecting the most geometrically representative triplets from the global set of all possible triplets, which restricts its achievable accuracy and stability in complex matching tasks.

Recently, fueled by the rapid development of artificial intelligence, deep learning-based feature matching algorithms such as SuperGlue [27] and LoFTR [28] have demonstrated remarkable performance gains. These methods typically employ graph neural networks or attention mechanisms to explicitly capture contextual dependencies among feature points, thus enhancing descriptor discriminability and improving matching accuracy. Under ideal conditions, where sufficient training data are available, computational resources are adequate, and the test scenarios are consistent with the training distribution, such methods can achieve high matching accuracy. However, the superior performance of deep learning-based matching methods represented by SuperGlue and LoFTR relies heavily on high-quality feature detection and description models trained on specific data distributions. In UAV remote sensing heterogeneous image matching tasks under GPS-denied environments, their computational cost and limited generalization capability significantly restrict their deployment on resource-constrained platforms. Specifically, onboard UAV computing platforms are typically subject to strict constraints in terms of computational power, memory, and energy consumption, making it difficult to support the complex forward inference of large-scale deep neural networks. Moreover, acquiring large-scale, pixel-level accurately annotated datasets that sufficiently cover the substantial imaging domain gap between satellite and UAV images is extremely costly, which poses severe domain generalization challenges for deep models. As a result, their performance may degrade dramatically in unseen scenes or under extreme viewpoint variations. More importantly, heterogeneous image matching is inherently characterized by a high mismatch rate. Even when using highly discriminative deep descriptors, the resulting initial correspondence sets often still contain a large number of outliers, and the final localization accuracy remains highly dependent on the performance of subsequent robust geometric estimation methods, such as RANSAC. Therefore, the core focus of this work is to enhance the robustness of geometric transformation estimation from initial correspondences heavily contaminated by outliers. The proposed method is designed to be compatible with various feature extractors and aims to provide an efficient and lightweight post-processing solution for addressing the common challenge of high outlier ratios in heterogeneous image matching. Accordingly, the comparative experiments in this paper are primarily conducted against advanced methods within the category of robust model fitting, in order to fairly evaluate the effectiveness of the proposed sampling strategy itself.

To balance the powerful representational capacity of deep learning methods with the generalization ability and efficiency of classical methods, a series of hybrid methods and unsupervised or self-supervised methods that do not heavily rely on annotated data have emerged in recent research. Hybrid methods attempt to integrate traditional geometric constraints (e.g., spatial consistency) with lightweight learning frameworks. For instance, by using neural networks to predict the confidence of matches to guide sampling [29], or by learning invariant features for local geometric transformations [30]. On the other hand, unsupervised methods leverage supervisory signals such as photometric consistency or cycle consistency between image pairs to learn matching priors from large amounts of unlabeled data [31,32]. These methods alleviate the dependency on pairwise annotated data to some extent and improve adaptability in cross-domain scenarios. However, in the specific mission of matching heterogeneous UAV remote sensing images, where imaging mechanisms differ significantly and viewpoints vary extremely, simple photometric constraints often fail, while learned local descriptors still face challenges in cross-domain generalization. Consequently, the robustness of the model fitting stage in the presence of a high proportion of outliers remains a prominent issue.

To overcome the limitations faced by traditional feature matching algorithms (e.g., RANSAC) in achieving stable matching between heterogeneous UAV and satellite images, which is challenging due to variations in feature distribution, scale, and illumination and consider the further shortcomings of existing methods. These methods, including classical random sampling, data-driven deep learning, and emerging hybrid or unsupervised approaches, still exhibit deficiencies in robustness and efficiency during model fitting when confronted with a high proportion of outliers. To address these issues, this paper proposes a spatial consistency-guided sampling algorithm to improve matching robustness and accuracy. Unlike earlier works that merely use triplets for one-time hypothesis generation, this study first constructs a globally comprehensive candidate triplet relation graph and designs a local geometric consistency filtering mechanism aimed at selecting high-confidence spatial structural units from the source. Subsequently, a minimal subset sampling strategy is designed to ensure that the sampled subsets are both representative and diverse in terms of global geometry. Building on this foundation, a data subset refinement strategy is introduced to improve the convergence efficiency of the sampling and model fitting processes. Therefore, the proposed method not only leverages the constraint capability of triplets but also embeds them into a complete, coarse-to-fine robust estimation pipeline. Ultimately, the estimated model hypotheses and parameters effectively distinguish inliers from outliers, enabling more stable and precise geometric model estimation for heterogeneous image registration missions.

The main innovations and contributions of this study can be summarized as follows:

(1) A spatial consistency-guided sampling algorithm is proposed, which constructs triplet relationships within feature point neighborhoods to characterize the stability of local geometric structures, thereby capturing structurally similar corresponding regions across heterogeneous images and improving the inlier probability and robustness of initial sampling.
(2) A minimal subset sampling strategy is proposed to improve the global coverage and geometric representativeness of heterogeneous image matching, thereby enhancing its overall robustness.
(3) A data subset refinement strategy based on iterative optimization is introduced to improve the convergence efficiency and matching accuracy of heterogeneous image matching.

The rest of the paper is structured as follows: Section 2 provides a detailed description of the proposed method; Section 3 presents the datasets and evaluation criteria; Section 4 reports and analyzes the experimental results; and Section 5 draws the conclusions.

## 2. The Proposed Sampling Algorithm

In feature matching, RANSAC is commonly employed to mitigate the influence of outliers generated in the initial feature extraction process. RANSAC refines the initially matched data to improve overall accuracy. However, when the input data contain a high proportion of outliers, RANSAC becomes computationally intensive and lacks sufficient spatial constraints, thereby limiting its ability to effectively eliminate outliers in heterogeneous image matching missions. To address this limitation, a spatial consistency-guided sampling algorithm is proposed to enhance the robustness and accuracy of feature matching. The algorithm first constructs a triplet relationship within the feature point set to characterize local geometric structures. By jointly analyzing the geometric relationships among neighboring points, triplets exhibiting both geometric stability and local consistency are extracted. Subsequently, a minimal subset sampling strategy is proposed, leveraging triplet scores and spatial distribution features to generate sampling subsets that are both globally representative and geometrically diverse. Building upon this, a data subset refinement strategy is introduced to improve the convergence efficiency of both the sampling and model fitting processes. Ultimately, the proposed algorithm derives optimal model hypotheses and parameters to effectively distinguish inliers from outliers, achieving stable and precise geometric model estimation for heterogeneous image registration.

### 2.1. Principle of Triplet Relationship

Traditional triplet relationship-guided sampling consensus for robust model estimation generally employs random sampling, selecting triplets randomly from the complete set of correspondences to form model hypotheses. By contrast, the key difference of our proposed triplet relationship is that triplets are not regarded merely as random units, but as fundamental building blocks for capturing local spatial structural consistency across images. The main objective of the proposed algorithm is to systematically form triplets over the input feature points that capture stable local geometric structures, serving as a high-quality initial candidate set for subsequent guided sampling.

The core idea of the proposed algorithm involves employing triplet relationships to guide the sampling of informative data subsets for robust estimation. Specifically, the algorithm first examines the triplet relationships among the input feature correspondences. Each triplet comprises three feature points that satisfy spatial consistency constraints, thereby forming a set of locally stable geometric structures.

**Figure 1:**
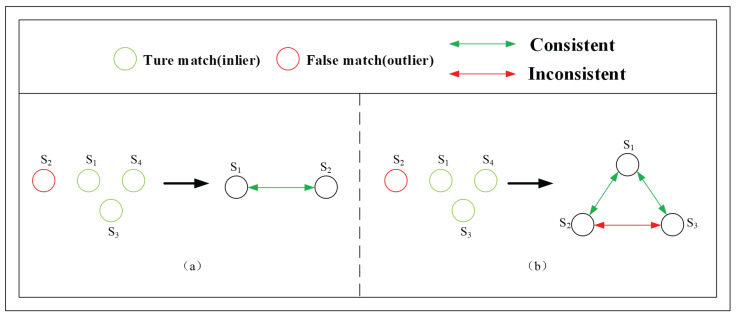
Comparison of robust model estimation using (**a**) pairwise relationships and (**b**) triplet relationships.

As illustrated in Figure 1, $s_{i}$ denotes the planar projection of a corresponding feature pair between two images. In Figure 1a, $s_{1}$ and $s_{2}$ satisfy the spatial consistency constraint because their corresponding features are spatially adjacent. Therefore, they are both identified as inliers. In Figure 1b, the triplet relationship imposes constraints on data points. Because $s_{2}$ is neighbored by $s_{1}$ and $s_{3}$, while $s_{3}$ is neighbored by $s_{1}$ and $s_{4}$, the pair $s_{2}$ and $s_{3}$ fails to meet the spatial consistency constraint, and thus $s_{2}$ is excluded as an inlier. Therefore, the multiple constraints embedded in the triplet can effectively detect outliers among the match sets.

For a pair of images with *N* feature correspondences $A={\left\{s_{i}\right\}}_{i=1}^{N}$, let $s_{i}=\left\{x_{i},y_{i}\right\}$ denote the correspondence set, where $x_{i}$ and $y_{i}$ are the pixel coordinates of the feature points in the two images, respectively. The correspondences between image pairs are obtained through feature extraction and description processes (e.g., SuperPoint [33] or SIFT [34]). According to the spatial relationships of feature points $s_{i}$, the *K*-nearest neighbor set $N_{s_{i}}$ is determined for each point. For a feature point $s_{i}$, its triplet relationship with other feature points in the set *A* is defined as follows:

A triplet $\Delta_{s_{i}s_{j}s_{k}}$ is composed of three feature points and defined as $\Delta_{s_{i}s_{j}s_{k}}=\left(s_{i},s_{j},s_{k}\right)$, if and only if $s_{i}$, $s_{j}$, and $s_{k}$ satisfy the following condition:

(1) $$1_{s_{i}\in N_{s_{j}}}\times 1_{s_{k}\in N_{s_{i}}}\times 1_{s_{j}\in N_{s_{k}}}=1$$

where $s_{i/k/j}\in N_{s_{j/i/k}}$ indicates that $s_{i/k/j}$ belongs to the *K*-nearest neighbor set of $s_{j/i/k}$, and the indicator function $1_{s_{i/k/j}\in N_{s_{j/i/k}}}$ equals 1 when the condition holds, and 0 otherwise. The parameter *K* defines the size of the local neighborhood for constructing triplet relationships. A small *K* may result in insufficient neighborhood information, preventing the capture of stable local geometric structures and increasing noise sensitivity; conversely, a large *K* may introduce excessive outliers into the neighborhood, weakening local structural consistency and raising computational overhead. To determine a suitable *K*, this work proposes a heuristic setting based on the density of local feature points. First, compute the Euclidean distances between all feature point pairs in the image to derive the average nearest-neighbor distance *d*. Then, *K* is set proportional to $N/(d\times S_{c})$ according to the image size $S_{c}$ and the number of feature matches *N*, and is experimentally constrained within an empirical range. In the experiments, it was observed that features in UAV–satellite heterogeneous image pairs are relatively sparse yet structurally significant, and setting *K* to 7 yields robust performance in most cases.

If feature point $s_{i}$ forms a triplet $\Delta_{s_{i}s_{j}s_{k}}$ with $s_{j}$ and $s_{k}$, these three feature correspondences are regarded as the triplet feature group associated with $s_{i}$. It is worth noting that in heterogeneous images, due to the presence of numerous outliers, $s_{i}$ may form multiple triplets with other points. That is, for feature point $s_{i}$, there may exist three or more points satisfying the above condition, thus forming multiple triplets. Based on the above analysis, the constructed triplets are represented as a new set $\chi =\{\Pi_{s_{i}}\}$, where $\Pi_{s_{i}}=\left(X_{i},Y_{i}\right)$ denotes the coordinate information of a triplet $\Delta_{s_{i}s_{j}s_{k}}$ in the two heterogeneous images.

Although the complementary constraints within triplets substantially reduce sensitivity to outliers, incorrect classifications may still arise when outliers are in close spatial proximity to inliers. To address this issue, a local geometric structure consistency filtering mechanism is subsequently applied to the constructed triplet feature vectors to retain only those triplets that adhere to local geometric coherence.

### 2.2. Local Geometric Structure Consistency Filtering

For a given set of triplets $\chi =\{\Pi_{s_{i}}\}$, their coordinate sets in the two images are defined as:

(2) $$X_{i}=\left[\begin{array}{c} \begin{array}{cc} x_{i} & \begin{array}{cc} x_{j} & x_{k} \end{array} \end{array}\\ \begin{array}{cc} y_{i} & \begin{array}{cc} y_{j} & y_{k} \end{array} \end{array} \end{array}\right],Y_{i}=\left[\begin{array}{c} \begin{array}{cc} x_{i}^{\prime } & \begin{array}{cc} x_{j}^{\prime } & x_{k}^{\prime } \end{array} \end{array}\\ \begin{array}{cc} y_{i}^{\prime } & \begin{array}{cc} y_{j}^{\prime } & y_{k}^{\prime } \end{array} \end{array} \end{array}\right],$$

From these coordinate sets, the centroids of each triplet can be computed as:

(3) $$c_{1}=\frac{1}{3}\sum_{i=1}^{3}X_{k},c_{2}=\frac{1}{3}\sum_{i=1}^{3}Y_{k}$$

The side lengths of each triplet are then defined as:

(4) $$L_{1}=\left[\begin{array}{c} {∥X_{i}-X_{j}∥}_{2}\\ {∥X_{j}-X_{k}∥}_{2}\\ {∥X_{k}-X_{i}∥}_{2} \end{array}\right],L_{2}=\left[\begin{array}{c} {∥Y_{i}-Y_{j}∥}_{2}\\ {∥Y_{j}-Y_{k}∥}_{2}\\ {∥Y_{k}-Y_{i}∥}_{2} \end{array}\right]$$

The feature vector of each triplet is constructed as:

(5) $$f_{i}={\left[c_{1},c_{2},L_{1},L_{2},g_{1},g_{2}\right]}^{T}$$

where $g_{1}$ and $g_{2}$ represent the sets of the three vertex angles of each triplet, calculated via the cosine law. Each triplet feature vector is then organized into a feature matrix:

(6) $$F=[f_{1},f_{2},\ldots ,f_{N}]$$

After obtaining the feature matrix that encodes the geometric properties of triplets, the local structural consistency between triplets can be evaluated. Specifically, for the *i*-th triplet and its neighbors $j\in N_{i}$, we define:

(7) $$l_{x}\left(j,i\right)=\left\{\begin{array}{cc} 1, & if\left|∥f_{i}∥-∥f_{j}∥\right|>\lambda ∥f_{i}∥\\ 0, & otherwise \end{array}\right.$$

Similarly, $l_{y}\left(j,i\right)$ can be obtained. The edge affinity between triplets is then defined as:

(8) $$S_{e}\left(j,i\right)=\frac{1}{K}\exp\left(-\frac{\left|d_{ij}-\Upsilon {\bar{d}}_{i}\right|}{\max(d_{ij},\Upsilon {\bar{d}}_{i})}\right)$$

where $d_{ij}={∥f_{i}-f_{j}∥}_{2}$, ${\bar{d}}_{i}=\frac{1}{K}\sum_{j\in N_{i}}d_{ij}$. The node affinity of each triplet is then computed as:

(9) $$S_{v}\left(i\right)=1-\frac{\sum_{j\in N_{i}}\left(l_{x}\left(j,i\right)+l_{y}\left(j,i\right)\right)}{2K}$$

Finally, a weighted structural consistency score is computed by jointly considering edge and node affinities:

(10) $$w\left(i\right)=\frac{1}{2}\left(S_{v}\left(i\right)+\frac{1}{K}\sum_{j\in N_{i}}S_{e}\left(j,i\right)\right)$$

A data-driven adaptive thresholding method is proposed to extract triplets with locally consistent structures from the set χ. Specifically, the consistency score set ${\left\{w\left(i\right)\right\}}_{i=1}^{N}$ of all candidate triplets χ is computed, and the mean $\mu_{s}$ and standard deviation $\sigma_{s}$ of this set are then determined. The threshold $\lambda_{t}$ is defined as $\lambda_{t}=\mu_{s}+\alpha \cdot \sigma_{s}$, where α is a tunable coefficient, which is set to 0.5 in all experiments presented here. This means that the algorithm preferentially keeps triplets whose consistency scores exceed the average. α governs the rigor of the filtering, with larger values imposing more stringent selection conditions. Based on the computed structural consistency weight, a binary function $p_{i}$ is established as follows:

(11) $$p_{i}=\left\{\begin{array}{cc} 1, & if\;w\left(i\right)>\lambda_{t}\\ 0, & otherwise \end{array}\right.$$

Thus, a series of binary vectors *P* can be obtained:

(12) $$P=[p_{1},p_{2},\ldots ,p_{N}]$$

Finally, the triplets exhibiting high local geometric consistency are preserved according to the binary selection vector:

(13) $$C={\left\{s_{i}|p_{i}=1\right\}}_{i=1}^{N}$$

### 2.3. A Minimal Subset Sampling Strategy

To effectively select the initial subset, this paper proposes a minimal subset sampling strategy, which further refines the previously filtered triplet groups to better identify potential inliers within the dataset. Specifically, after obtaining the new triplets $C={\left\{s_{i}|p_{i}=1\right\}}_{i=1}^{N}$, their compatibility scores can be computed based on pairwise relationships:

(14) $$f\left(s_{i},s_{j}\right)=\exp\left(-{\left({∥y_{j}-y_{i}∥}_{2}-{∥x_{j}-x_{i}∥}_{2}\right)}^{2}\right)$$

A pair of feature matches that satisfy the spatial consistency constraint in Equation (14) can obtain a high compatibility score and are therefore considered to have a high inlier probability. However, computing the compatibility score based solely on pairwise relationships may not be robust enough against outliers, especially when numerous outliers exist in heterogeneous images. To enhance robustness against outliers, the triplet relationship is integrated into the compatibility computation process:

(15) $$w\left(s_{i},s_{j},s_{k}\right)=f\left(s_{i},s_{j}\right)+f\left(s_{j},s_{k}\right)+f\left(s_{k},s_{i}\right)$$

As described in Equation (15), a high compatibility score indicates that all three correspondences within a triplet are inliers; otherwise, the score remains low. Then, all triplet compatibility scores are represented as a score vector $W=[w_{1},w_{2},\ldots ,w_{N}]$, and the scores are normalized as $W_{i}^{\prime }=W_{i}-\min\left(W\right)$ to obtain the initial sampling probability distribution:

(16) $$p_{i}=\frac{W_{i}^{\prime }}{\sum_{j=1}^{N}W_{j}^{\prime }}$$

For each candidate feature $s_{i}$, its Euclidean distance $d_{j,i_{t}}$ to already selected samples $s_{i_{t}}$ is computed, To adaptively set the strength of spatial constraints, we associate the Gaussian kernel variance $\sigma^{2}$ with the spatial distribution of feature points in the current image pair. Specifically, the Euclidean distances between all matching feature point pairs are computed, and the median is taken, denoted as $d_{med}$. Subsequently, $\sigma^{2}$ is assigned as $\sigma^{2}={\left(d_{med}\right)}^{2}$, followed by the Gaussian-kernel-based diversity penalty term:

(17) $$\emptyset_{j,i_{t}}=1-\exp\left(-\frac{d_{j,i_{t}}^{2}}{2\sigma^{2}}\right)$$

where $\sigma^{2}$ denotes an empirically set variance parameter. Then, the sampling distribution is updated by combining the matching score with the diversity constraint:

(18) $$p_{i}=\frac{W_{i}^{\prime }\cdot \emptyset_{j,i_{t}}}{\sum_{j=1}^{N}W_{j}^{\prime }\cdot \emptyset_{j,i_{t}}}$$

After iterating for *t* rounds, the strategy converges to the desired minimal sampling subset. The proposed minimal subset sampling strategy effectively selects representative points to form reliable model hypotheses, mitigating the impact of outliers and enabling fast generation of clean, robust subsets for estimation.

The minimal subset sampling strategy helps prevent selecting most outliers during the sampling process. However, outliers may still be included in a data subset, especially when the input feature correspondences contain numerous outliers, leading to invalid subsets for model estimation. Hence, a data subset refinement strategy is further introduced to progressively extract a sufficient number of inliers and construct a valid, high-quality subset for model estimation.

### 2.4. Data Subset Refinement Strategy

**Figure 2:**
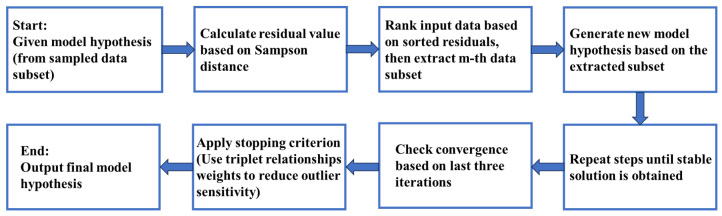
Design process of the data subset refinement strategy.

For a given model hypothesis $\upsilon_{\lambda }$ generated from a sampled data subset, the residuals ${\left\{r\left(s_{i},\upsilon_{\lambda }\right)\right\}}_{i=1}^{N}$ between the hypothesis $\upsilon_{\lambda }$ and the input data $A={\left\{s_{i}\right\}}_{i=1}^{N}$ are calculated using the Sampson distance. Next, the residuals are sorted in ascending order, and a new model hypothesis is generated by extracting a data subset around the *n*-th data point according to the sorted residuals. These steps are repeated iteratively until convergence to a stable solution. Convergence of the data subset refinement process is assessed using the last three iterations, with MaxIter specifying the maximum number of iterations, and the weights computed from the sampled triplets serving as a stopping criterion to reduce sensitivity to outliers. Specifically, the stopping criterion $\Gamma_{stop}$ is defined as:

(19) $$\Gamma_{stop}=\left(\frac{1}{h}\sum_{j=n-h+1}^{n}w(s_{j}^{\upsilon_{\left(\lambda -1\right)}})<w\left(s_{n}^{\upsilon_{\lambda }}\right)\right)\wedge \left(\frac{1}{h}\sum_{j=n-h+1}^{n}w(s_{j}^{\upsilon_{\left(\lambda -2\right)}})<w\left(s_{n}^{\upsilon_{\lambda }}\right)\right)$$

where $s_{n}^{\upsilon_{\lambda }}$ denotes the *n*-th reference point in the sorted residuals at the λ-th iteration; ω(·) is the compatibility score computed from the triplets; and *h* is the size of the data subset [35]. This criterion indicates that if the sampled data subsets from the last three iterations are likely to belong to the same model, the iteration is terminated. In this way, the performance of data subset sampling is further improved, yielding a valid subset and generating a prominent model hypothesis for estimation. Finally, outliers and inliers can be identified based on the estimated model. The design process of the data subset refinement strategy is shown in Figure 2.

## 3. Datasets and Evaluation Metrics

To evaluate the effectiveness of the proposed algorithm, three challenging public UAV heterogeneous image datasets (University-1652, DenseUAV) are used to conduct comparative experiments. The comparative methods included RANSAC, LPM [36], TRESAC, and LGSC [37], as well as the proposed algorithm. All experiments are conducted on an Intel(R) Core(TM) i5-1035G1 CPU @1.00 GHz with 8 GB of RAM using MATLAB R2018b on a 64-bit Windows 10 operating system.

### 3.1. Datasets

**Figure 3:**
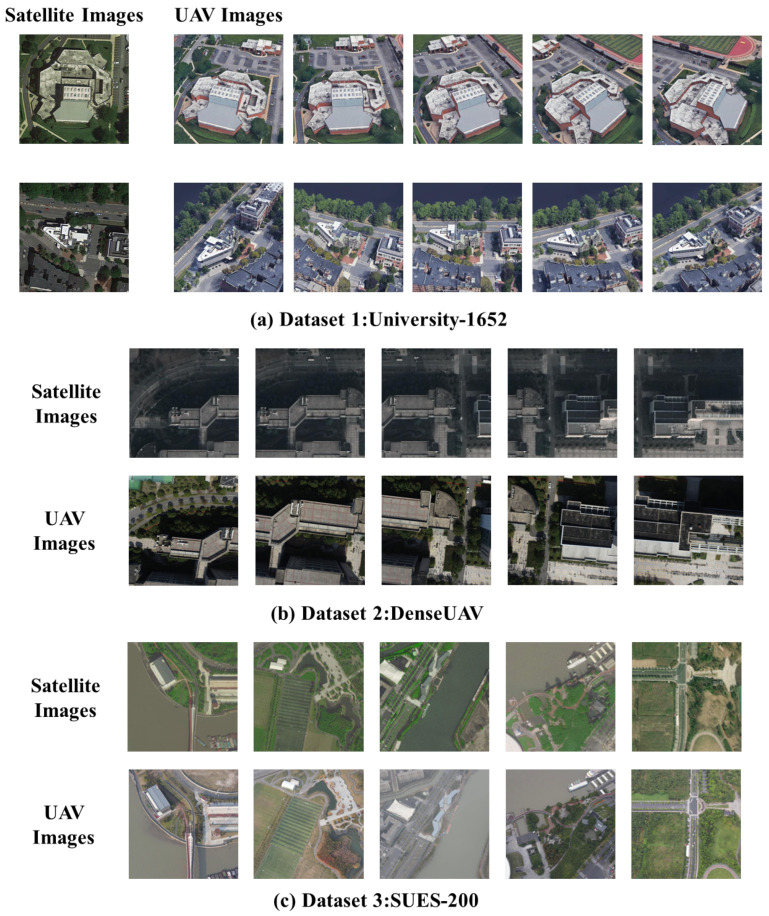
Datasets: (**a**) presents a selection of satellite images from the University dataset and their corresponding UAV images with oblique views. (**b**) illustrates portions of consecutive-frame satellite images from the DenseUAV dataset along with their corresponding UAV images. (**c**) shows images from rural areas and water body scenes in the SUES-200 dataset and the corresponding satellite images.

Dataset 1 corresponds to the publicly available University-1652 dataset, which has been widely employed in studies on visual localization and cross-view image retrieval. It contains images of 1652 building instances from 72 universities worldwide, encompassing three modalities: satellite views, UAV views, and street views. In the experiments, satellite images obtained from Google Maps and UAV images simulated from Google Earth viewpoints are primarily employed as matching targets. Each building instance offers one satellite view as an orthophoto reference and approximately 50 UAV images from different flight altitudes and viewpoints to facilitate matching evaluation, as shown in Figure 3a [38].

Dataset 2 corresponds to the publicly available DenseUAV dataset, which represents the first dataset specifically designed for UAV self-localization tasks. DenseUAV conducted dense data acquisition of UAV images in low-altitude urban environments, gathering and annotating over 27,000 UAV and satellite images from 14 university campuses, thereby establishing a new benchmark dataset. This dataset is utilized to assess the robustness and performance of the proposed algorithm in UAV and satellite image matching tasks, as illustrated in Figure 3b [39].

Dataset 3 corresponds to the publicly available SUES-200 dataset, which contains 24,120 UAV-acquired images at various altitudes and their corresponding satellite images of the same target scenes. This dataset encompasses scenes from rural areas, forests, and water bodies, and under varying weather conditions, enabling effective validation of the algorithm’s robustness to substantial appearance variations encountered in practical UAV missions, as illustrated in Figure 3c [40].

### 3.2. Evaluation Metrics

To comprehensively assess the feature matching performance of the proposed method and compare it with other state-of-the-art algorithms to validate its effectiveness and superiority, four evaluation metrics are employed. The evaluation metrics include precision, recall, and F-score [41] and mean projection error.

(1) Precision

Precision is used to quantify the proportion of correctly matched points among the detected inliers, with the formula:

(20) $$\mathrm{precision}=\frac{t_{p}}{t_{p}+f_{p}}$$

where $t_{p}$ represents the number of points correctly classified as inliers, and $f_{p}$ represents the number of points incorrectly classified as inliers.

(2) Recall

Recall quantifies the proportion of detected inliers relative to the ground-truth inliers, with the formula:

(21) $$\mathrm{recall}=\frac{t_{p}}{t_{p}+f_{n}}$$

where $f_{n}$ is the number of inliers mistakenly classified as outliers. A higher recall indicates that more inliers are retained.

(3) F-score

To simultaneously consider precision and recall, the F-score is employed to assess the comprehensive matching performance, with the formula:

(22) $$F\text{-}\mathrm{score}=\frac{2\times \mathrm{precision}\times \mathrm{recall}}{\mathrm{precision}+\mathrm{recall}}$$

(4) Mean Projection Error (MPE)

To assess the spatial accuracy of the estimated geometric model, the mean projection error (MPE) is employed. This metric calculates the mean reprojection error, in pixels, of all inlier points under the final estimated homography on their corresponding images. The lower the MPE, the higher the geometric alignment accuracy of the model.

## 4. Experimental Results and Analysis

To comprehensively evaluate the effectiveness and generality of the proposed spatial coherence-guided sampling feature matching algorithm, a series of systematic comparative experiments are conducted. The experiments cover algorithm parameter analysis and discussion, ablation studies, and matching performance across various scenarios, and performed using the public University-1652 heterogeneous matching dataset, the DenseUAV self-localization dataset, and the SUES-200 dataset.

### 4.1. Experiment 1—Parameter Analysis and Discussion

**Figure 4:**
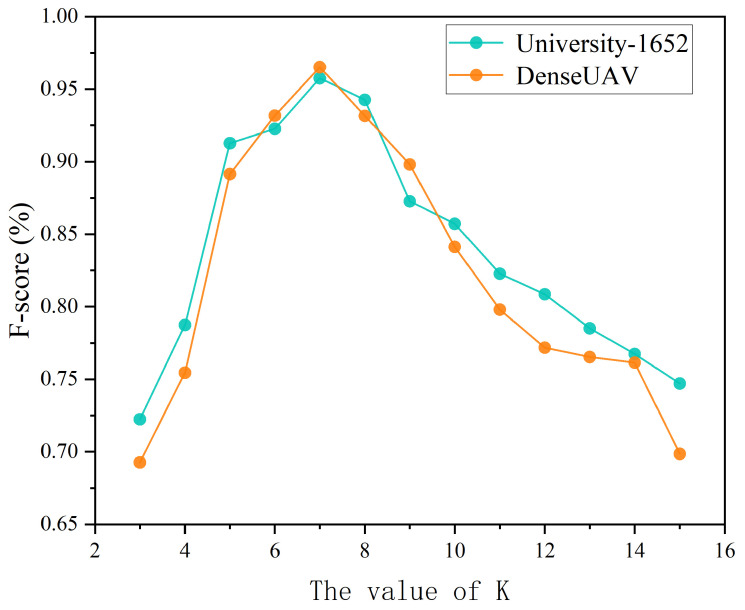
Effect of different *K* values on matching F-score.

To assess the robustness of the proposed algorithm to the key parameter *K*, sensitivity experiments are performed on selected subsets of the University-1652 and DenseUAV datasets. Keeping all other parameters and procedures fixed, only *K* is varied to observe its effect on the matching F-score. The results are presented in Figure 4.

The experiments show that the proposed algorithm maintains relatively stable and high performance when *K* is in the range [6, 8]. When *K* is too low (<6), performance drops due to overly strict neighborhood constraints, which hinder the formation of enough triplets for reliable filtering. Performance slightly decreases when *K* exceeds 8, as the neighborhood includes excessive noisy points that compromise local structural consistency. This confirms the rationality of selecting K=7 and indicates that the algorithm is robust within a reasonable neighborhood around this *K* value.

### 4.2. Experiment 2—Ablation Study

To assess the computational overhead and matching performance of the proposed data subset refinement strategy, we conduct an ablation study in this section. On a 50-image-pair test subset of the University-1652 dataset, we compare two configurations: (A) using only the basic triplet-guided sampling (without the data subset refinement strategy), and (B) the full algorithm (including the data subset refinement strategy). The experiment measures the average F-score, runtime, and the time specifically consumed by the refinement strategy, as summarized in Table 1.

As shown in Table 1, incorporating the data subset refinement strategy increases the F-score by 24.72 percentage points. At the same time, the runtime increases by about 11.72%, with an extra 0.026 s incurred. For applications requiring real-time UAV visual localization, the additional processing time is acceptable, particularly given the significant enhancement in matching accuracy, which directly impacts localization reliability. Compared with the costs arising from localization failures due to incorrect matches or the need for more complex post-processing, this overhead is justified. In addition, the overall algorithm remains a lightweight geometric post-processing procedure, with runtime significantly lower than the forward inference time of deep learning-based end-to-end matching networks, demonstrating its applicability on resource-constrained platforms.

### 4.3. Experiment 3—University-1652 Dataset

To validate the robustness and performance advantages of the proposed matching algorithm, and to assess its capability in achieving effective matching between UAV images and satellite images captured from varying perspectives in visual localization and navigation missions, the experiment employs UAV images with diverse oblique angles along with their corresponding satellite images from the University-1652 dataset. Comparative evaluations are conducted across multiple feature matching algorithms, with performance metrics summarized in Table 2.

**Figure 5:**
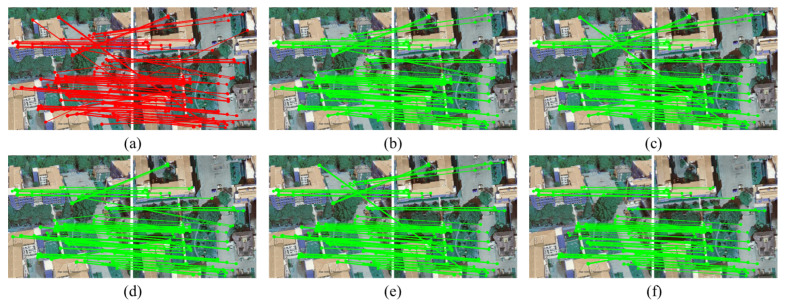
Partial matching outcomes of various algorithms on the University-1652 dataset. Panels (**a**–**f**) show: initial matches from feature extraction, RANSAC, LPM, TRESAC, LGSC, and our proposed method, respectively.

Figure 5 illustrates partial matching results of different algorithms on the University-1652 dataset. As shown in Figure 5 and Table 2, following outlier removal, RANSAC attains a precision of 79.81%, a recall of 98.78%, and an F-score of 87.87%, indicating that RANSAC exhibits limited capability in suppressing noise under high outlier ratios, leading to significant deviations in estimated models. While LPM achieves a relatively high recall of 93.63%, its precision is only 83.87%, implying a tendency to erroneously discard certain true inliers in heterogeneous images with complex geometric structures. In contrast, the proposed algorithm maintains a recall of 88.97% while achieving a precision of 91.11% and an F-score of 89.66%, substantially outperforming the comparative algorithms, thereby achieving a more favorable balance between inlier retention and outlier removal.

As shown in Table 2, compared with traditional TRESAC and LGSC, the proposed algorithm exhibits enhanced robustness against outliers. In scenarios with a high proportion of outliers, TRESAC achieves a precision of only 84.24%, whereas the proposed algorithm reaches 91.11%, an improvement of approximately 8.2%. Additionally, compared with LGSC, the proposed method demonstrates superior overall performance, with the F-score increasing from 88.71% to 89.66%, indicating enhanced stability and generalization under complex heterogeneous image conditions. Moreover, the mean projection error achieved 0.0711 pixels, considerably lower than that of RANSAC and LGSC. This demonstrates that the proposed algorithm is capable of selecting a larger number of correct correspondences and leveraging them to estimate a more precise spatial transformation, which is critical for achieving high final accuracy in UAV visual localization. This improvement is attributed to the minimal subset sampling strategy, which integrates both matching scores and spatial distribution constraints derived from the consistency of local neighborhood structures. The spatially diverse sampling strategy guided by triplet relations effectively enhances the representativeness and distribution balance of candidate samples. Furthermore, during the subsequent data subset refinement phase, a data subset refinement strategy is implemented, facilitating rapid convergence of geometric model estimation to a stable solution under high-outlier conditions, further enhancing the accuracy and robustness of feature matching.

### 4.4. Experiment 4—DenseUAV Dataset

To further validate the adaptability and practical applicability of the proposed algorithm in UAV self-localization tasks under complex conditions, the DenseUAV dataset is utilized for experimental validation. Unlike the University-1652 dataset, which primarily targets heterogeneous ground-view matching, DenseUAV is the first publicly available dataset comprising high-density, low-altitude aerial image sequences specifically designed for UAV self-localization missions. The aerial images in this dataset generally exhibit noticeable viewpoint drift, illumination changes, and large areas of similar textures, thereby providing a more realistic representation of UAV continuous navigation and localization in urban canyons or densely built areas where GPS signals are often obstructed.

**Figure 6:**
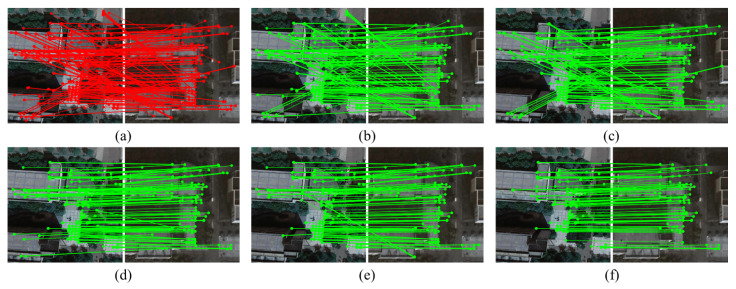
Partial matching outcomes of various algorithms on the DenseUAV dataset. Panels (**a**–**f**) show: initial matches from feature extraction, RANSAC, LPM, TRESAC, LGSC, and our proposed method, respectively.

In the experiments, multiple sequences of consecutive frames and their corresponding satellite reference images are extracted from the DenseUAV dataset for matching evaluations, with some results shown in Figure 6. To comprehensively compare the performance of different algorithms on large-scale aerial data, mainstream robust model fitting algorithms, including RANSAC, LPM, LGSC, and TRESAC, are employed for comparative analysis, assessing their effectiveness in outlier removal, matching accuracy, and robustness.

As shown in Table 3, for large-scale image sequences with significant viewpoint variations, RANSAC exhibits insufficient robustness in outlier removal, achieving a precision of 75.43%, a recall of 98.38%, and an F-score of 84.92%. LPM attains a high recall of 95.68%, yet its precision is only 77.97%, indicating that excessive dependence on local neighborhood structures may lead to erroneous exclusion of true inliers. TRESAC and LGSC demonstrate improvements over conventional approaches, with LGSC achieving a better balance between precision and recall at 88.86% and 89.50%, respectively, although overall performance remains slightly inferior to the proposed method.

In contrast, the proposed algorithm demonstrates superior overall performance on the DenseUAV dataset, with a precision of 90.03%, recall of 89.10%, and an F-score of 88.80%, effectively identifying corresponding feature points under complex lighting and viewpoint variations. The proposed algorithm retains more valid inliers while effectively suppressing outlier interference caused by viewpoint shifts in consecutive frames and dynamic background changes, thereby achieving more accurate UAV–satellite image registration.

Experimental results on the DenseUAV dataset strongly corroborate the effectiveness of the proposed approach in UAV self-localization missions. By incorporating a minimal subset sampling strategy and a data subset refinement strategy, the proposed algorithm significantly enhances the stability of heterogeneous image matching and the robustness of localization, further corroborating its generalization capability and practical applicability in complex urban environments.

### 4.5. Experiment 5—SUES-200 Dataset

**Figure 7:**
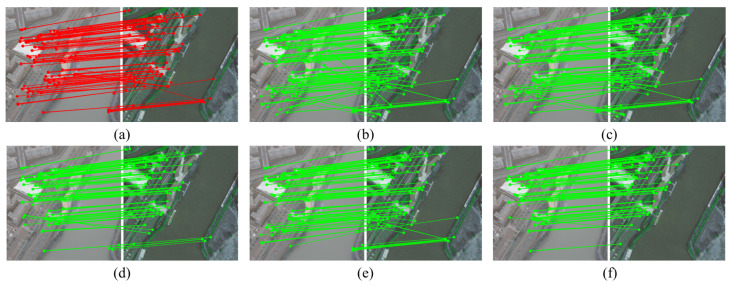
Partial matching outcomes of various algorithms on the SUES-200 dataset. Panels (**a**–**f**) show: initial matches from feature extraction, RANSAC, LPM, TRESAC, LGSC, and our proposed method, respectively.

To assess the robustness and generalization of the proposed algorithm in more diverse and heterogeneous real-world scenarios, experiments are conducted on the SUES-200 dataset, comprising rural and natural environments. This dataset comprises UAV-acquired images paired with corresponding satellite imagery, covering rural areas, forests, water bodies, and scenes under different weather conditions, allowing effective evaluation of the algorithm’s robustness to substantial appearance changes in practical UAV missions. Partial results are illustrated in Figure 7.

Table 4 shows the performance of the comparative algorithms on this dataset. Consistent with the results on the University-1652 and DenseUAV datasets, our algorithm continues to demonstrate the best overall performance, with an F-score of 92.65%, markedly superior to other algorithms. Importantly, our algorithm attains the lowest mean projection error of 0.0463 pixels, demonstrating that it can estimate highly accurate geometric transformations even in natural scenes with significant appearance changes. By comparison, RANSAC and TRESAC show markedly higher MPE and substantially lower recall, illustrating their limitations in addressing feature matching ambiguities in these scenarios.

### 4.6. Experiment 6—Statistical Significance Test

To assess the statistical stability of the proposed algorithm, 50 randomly selected image pairs from each of the three datasets are independently matched in repeated trials. All competing algorithms are executed on the same image pairs, recording precision, recall, and F-score for each run. The final reported performance metrics represent the mean ± standard deviation across multiple trials. To quantify the statistical significance of performance differences, a two-tailed paired *t*-test is conducted with a significance level of 0.05. If the *p*-value of a comparison between a baseline algorithm and our method is below 0.05, the performance difference is deemed statistically significant.

Table 5 shows the results of the statistical significance tests for all compared algorithms. Compared to RANSAC, the proposed algorithm demonstrates a positive trend on all three datasets, reaching statistical significance on University-1652 and SUES-200, and marginal significance on DenseUAV. This confirms the superiority of the proposed guided sampling strategy relative to traditional random sampling. Compared with LPM, the proposed algorithm shows a positive trend across all three datasets, achieving statistical significance on two datasets and marginal significance on University-1652. This demonstrates that the proposed algorithm enhances matching precision while preserving a high recall rate. Compared with LGSC, the proposed algorithm demonstrates statistical significance on University-1652 and DenseUAV datasets, but not on the SUES dataset. This indicates that the proposed algorithm generally outperforms the LGSC method. Compared to TRESAC, the proposed algorithm demonstrates a positive trend across all three datasets, showing extreme significance on University-1652. This indicates that the proposed algorithm generally outperforms the traditional triplet relationship-guided sampling consensus algorithm.

### 4.7. Discussion

**Figure 8:**
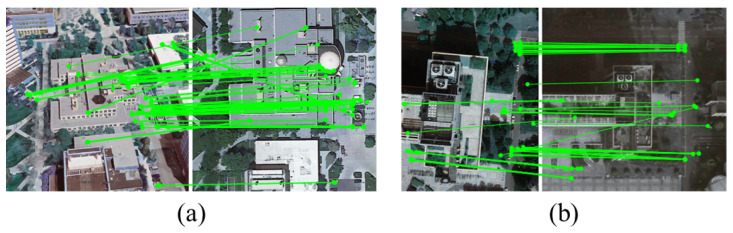
Two erroneous matches produced by the proposed method on two datasets: (**a**) an image pair from the University-1652 dataset; (**b**) an image pair from the DenseUAV dataset.

The proposed spatial consistency-guided sampling algorithm demonstrates excellent robustness in UAV remote sensing heterogeneous image matching. In practical scenarios, the proposed algorithm performs well on challenging datasets that are severely affected by outliers. For instance, applying the method to the image pairs in Figure 5 and Figure 6 yields F-scores of 93.80% and 93.33%, respectively. This performance is primarily due to the three core components of the proposed method: first, constructing and filtering triplet relationships that encode local geometric structures, enhancing spatial consistency constraints during sampling; in addition, using a guided sampling strategy that combines scoring and diversity to ensure geometric representativeness of the initial subset; and finally, applying the data subset refinement strategy to further improve convergence accuracy and stability in model estimation. However, it is noteworthy that the proposed algorithm still has limitations when dealing with certain extremely challenging scenarios. For instance, the initial inlier ratios of the image pairs in Figure 8a and Figure 8b are 43.39% and 46.07%, respectively. Although the proposed algorithm strengthens filtering via triplet relationships, the fundamental lack of reliable geometric structures results in final matching precisions of only about 72.06% and 73.83%, far below average. This highlights the proposed algorithm’s dependence on the quality of the base matches provided by feature extraction, and the input data must contain a sufficient number of inliers whose local spatial structures remain stable or approximately stable across views. The proposed algorithm’s performance deteriorates when local geometric deformations exceed the approximate range of similarity transformations. This understanding delineates the applicable scope of the current method and suggests directions for future research toward more adaptive improvements, such as investigating relational representations that are invariant to more complex geometric transformations.

## 5. Conclusions

This paper addresses the problem that traditional feature matching algorithms demonstrate limited robustness and unstable model fitting performance in scenarios with numerous outliers, arising from image heterogeneity and variations in viewpoints during UAV autonomous localization and navigation missions. A spatial consistency-guided sampling algorithm is proposed. The proposed algorithm models spatial consistency by establishing a triplet relationship within local neighborhoods, thereby enhancing the stability and structural comparability of geometric features in heterogeneous images while generating a high-confidence initial subset for subsequent matching. In the initial subset selection stage, a minimal subset sampling strategy is proposed. This strategy incorporates a normalized matching score and spatial distribution constraints to ensure that the sampled subset maintains both geometric representativeness and spatial uniformity globally, thus preventing feature clustering and local redundancy typical of conventional random sampling, significantly enhancing global matching stability and generalization capability. For the inlier optimization stage, a data subset refinement strategy is further introduced to effectively mitigate model overfitting and outlier effects. To demonstrate the effectiveness of the proposed algorithm, systematic comparative experiments are conducted on the University-1652 heterogeneous matching dataset and the DenseUAV UAV self-localization dataset. The results show that the proposed algorithm markedly surpasses traditional RANSAC and TRESAC in terms of outlier removal, matching accuracy, and convergence stability, thereby demonstrating its robustness and practical utility. In conclusion, the spatial consistency-guided sampling algorithm presented herein provides an effective, efficient, and broadly generalizable solution for UAV visual autonomous localization and heterogeneous image registration missions.

However, the proposed algorithm may face challenges under more complex conditions. First, when there are independently moving objects in the scene, such as vehicles or pedestrians, the feature points on these objects form ’structural outliers.’ While these moving feature points may locally satisfy geometric consistency, their motion violates the global spatial constraints relative to the static points. The proposed algorithm might find it challenging to differentiate such moving object clusters from the static background, potentially resulting in the model fitting to an incorrect moving object plane. Secondly, when the matching data originates from sensors with larger imaging modality differences, such as RGB images, infrared imagery, or LiDAR point clouds, extracting and describing feature points becomes inherently more difficult. The local geometric structures underpinning the triplet relationships may not directly align in feature spaces across different modalities, causing relationship metrics computed from single-image plane geometry to become invalid. To address these challenges, future work will explore incorporating temporal information or motion priors in dynamic environments, extending single-frame spatial consistency constraints into spatiotemporal constraints to remove feature point clusters induced by independent motion. Secondly, for multi-source data, more tightly constrained feature relationship metrics could be developed and combined with the proposed algorithm, with the goal of improving the autonomy and global positioning accuracy of UAV navigation systems in more complex, realistic environments.

## Figures and Tables

**Table 1:** Ablation Analysis of the Data Subset Refinement Strategy.

Configuration	F-Score (%)	Runtime (s)	Time for Refinement Strategy (s)
A: Basic Sampling	67.77	0.1958	0
B: Full Algorithm	92.49	0.2218	0.026

**Table 2:** Matching performance of different algorithms on the University-1652 dataset.

Metric	RANSAC	LPM	LGSC	TRESAC	The Proposed Algorithm
Precision (%)	79.81	83.87	88.35	84.24	91.11
Recall (%)	98.78	93.63	90.07	83.97	88.97
F-score (%)	87.87	88.01	88.71	83.32	89.66
Runtime (s)	1.5112	0.0179	0.0324	0.2172	0.1549
MPE (pixel)	0.1266	0.1025	0.0938	0.0819	0.0711

**Table 3:** Matching performance of different algorithms on the DenseUAV dataset.

Metric	RANSAC	LPM	LGSC	TRESAC	The Proposed Algorithm
Precision (%)	75.43	77.97	88.86	80.15	90.03
Recall (%)	98.38	95.68	89.50	87.53	89.10
F-score (%)	84.92	85.39	88.47	82.97	88.79
Runtime (s)	1.4931	0.0129	0.0303	0.2087	0.1517
MPE (pixel)	0.1165	0.1029	0.0907	0.0659	0.0513

**Table 4:** Matching performance of different algorithms on the SUES-200 dataset.

Metric	RANSAC	LPM	LGSC	TRESAC	The Proposed Algorithm
Precision (%)	80.41	86.63	91.25	89.21	94.63
Recall (%)	94.07	94.62	91.03	90.77	91.75
F-score (%)	86.10	90.30	91.88	89.64	92.65
Runtime (s)	1.4687	0.0084	0.0311	0.3184	0.1487
MPE (pixel)	0.1041	0.0977	0.0872	0.0597	0.0463

**Table 5:** Comparison of different methods across datasets.

Datasets	Sample Size	vs. RANSAC	vs. LPM	vs. LGSC	vs. TRESAC
University-1652	50	Significant (p=0.002)	Marginally significant (p=0.0545)	Marginally significant (p=0.0521)	Highly significant (p<0.001)
DenseUAV	50	Marginally significant (p=0.077)	Significant (p=0.016)	Significant (p=0.011)	Significant (p=0.031)
SUES-200	50	Significant (p=0.018)	Significant (p=0.013)	Not significant (p=0.331)	Marginally significant (p=0.081)
Trend/Consistency	–	Consistently positive (3/3)	Consistently positive (3/3)	Mostly positive (3/3)	Consistently positive (3/3)

## Data Availability

Data are contained within the article.
